# Tuning 2,3-Bis(arylimino)butane-nickel Precatalysts for High-Molecular-Weight Polyethylene Elastomers

**DOI:** 10.3390/molecules30081847

**Published:** 2025-04-20

**Authors:** Dongzhi Zhu, Dedong Jia, Qiuyue Zhang, Yanping Ma, Qaiser Mahmood, Wen-Hua Sun

**Affiliations:** 1 Guangxi Key Laboratory of Advanced Structural Materials and Carbon Neutralization, School of Materials and Environment, Guangxi Minzu University, Nanning 530105, China; dzzhu1992@iccas.ac.cn; 2 Key Laboratory of Engineering Plastics, Beijing National Laboratory for Molecular Sciences, Institute of Chemistry Chinese Academy of Sciences, Beijing 100190, China; jiaded@ahut.edu.cn (D.J.); zhangqiuyue@iccas.ac.cn (Q.Z.); 3 Chemistry and Chemical Engineering Guangdong Laboratory, Shantou 515031, China; qaiser@ccelab.com.cn

**Keywords:** unsymmetrical 2,3-bis(arylimino)butane-nickel complexes, structural modifications, high molecular weights, polyethylene elastomers

## Abstract

The catalytic performance of α-diiminonickel complexes is highly sensitive to structural modifications in their ligand frameworks. In this study, a series of unsymmetrical 2,3-bis(arylimino)butane-nickel complexes featuring *ortho*-2,6-dibenzhydryl groups as sterically demanding motifs and *para*-methyl groups as electron-donating enhancers were proposed and synthesized. These nickel complexes were thoroughly characterized using FTIR, elemental analysis, and single-crystal X-ray diffraction (for **Ni4** and **Ni5**), revealing deviations from ideal tetrahedral geometry. Upon activation with Et_2_AlCl, these complexes demonstrated exceptional ethylene polymerization activity, achieving a remarkable value of 13.67 × 10^6^ g PE mol^−1^ (Ni) h^−1^ at 20 °C. Notably, even at 80 °C, the nickel complexes maintained a high activity of 1.97 × 10^6^ g PE mol^−1^ (Ni) h^−1^, showcasing superiority compared to previously reported unsymmetrical 2,3-bis(arylimino)butane-nickel complexes. The resulting polyethylenes exhibited ultra-high molecular weights (*M*_w_: 3.33–19.47 × 10^5^ g mol^−1^) and tunable branching densities (84–217/1000C), which were effectively controlled by polymerization temperature. Moreover, the mechanical properties of the polyethylenes, including tensile strength (*σ*_b_ = 0.74–16.83 MPa), elongation at break (*ε*_b_ = 271–475%), and elastic recovery (SR = 42–74%), were finely tailored by optimizing molecular weight, crystallinity, and branching degree. The prepared polyethylenes displayed outstanding elastic recovery, a hallmark of high-performance thermoplastic elastomers, making them promising candidates for advanced material applications.

## 1. Introduction

Thermoplastic elastomers (TPEs), combining the flexibility of elastomers with the processability of thermoplastics [1,2,3,4,5,6,7,8], have emerged as cost-effective alternatives to conventional thermoset elastomers [9,10,11,12,13,14,15,16]. Traditional high-performance TPEs, such as ethylene/propylene copolymers and polyolefin elastomers, rely on multistep copolymerization with expensive α-olefins—a process fraught with complexity and high costs [17,18,19,20,21,22,23,24]. A significant shift occurred in 1995 with Brookhart’s groundbreaking α-diimine nickel/palladium catalysts, which utilize ethylene as the sole monomer to produce polyethylene with tunable branching density and branch type via a unique chain-walking mechanism [25,26]. Recent advances in ligand design of these catalysts have enabled the synthesis of branched polyethylene elastomers (PEEs) with tailored mechanical and thermal properties, positioning PEEs as promising TPE alternatives [27,28,29,30,31,32,33,34,35].

**Scheme 1:**
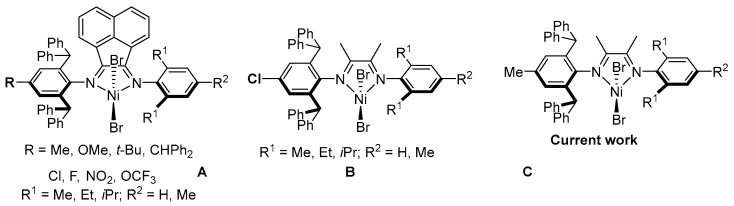
Steric and electronic variations in α-diimine nickel precatalysts **A** and **B,** along with this work **C**.

The mechanical performance of PEE hinges critically on molecular weight (*M*_w_), branching density, and crystallinity—properties governed by chain-walking and chain-transfer behaviors. Rational ligand design, focusing on synergistic steric and electronic modulation, has proven pivotal in enhancing thermal stability, suppressing β-H elimination, and finely tailoring polymeric microstructures [36,37,38]. Specifically, the introduction of ortho-dibenzhydryl groups into the N-aryl ring (**A**, Scheme 1) could significantly boost the thermal stability of nickel complexes and *M*_w_ of resulting polyethylenes [36,37,38,39,40,41,42,43]. Additionally, the electronic properties of *para*-substituents (electron-donating vs. electron-withdrawing) on the N-aryl ring were found to directionally regulate activity and branching density [36,37,38,39,40,41,42,43]. Electron-withdrawing groups (e.g., Cl [40], F [43], NO_2_ [41], OCF_3_ [38]) reduce the electron density at the metal center, accelerating ethylene coordination and insertion rates to achieve higher polyethylene molecular weights (*M*_w_), a result also supported by theoretical calculations [44]. In contrast, electron-donating groups (e.g., Me [42], OMe [39], *t*-Bu [37], CHPh_2_ [36]) enhance catalytic stability, promoting both chain propagation and chain-walking processes, which collectively elevate polymerization activity and branching degree. Remarkably, regarding the *para-*Me-substituent [42], it not only showed high activity (1.48 × 10^7^ g PE mol^−1^ (Ni) h^−1^) but also an extremely high branching degree, reaching 337/1000C. In contrast, for the *para*-Cl-substituent [40], the branching degree was 160/1000C. Evidently, the introduction of the electron-donating *para-*Me-substituent has played a crucial role in regulating the branching degree of the polyethylenes. Beyond substituent effects, the α-diimine backbone’s rigidity profoundly impacts catalytic behavior [45,46,47,48,49,50,51,52]. Our systematic studies revealed that 2,3-bis(arylimino)butane nickel complexes (**B**, Scheme 1) [52] yielded high-*M*_w_ polyethylenes with bimodal distributions, whereas their 1,2-bis(imino)acenaphthene analogues favored low-*M*_w_ unimodal products (**A_Cl_**, Scheme 1) [40]. This divergence underscores the backbone’s role in balancing conjugation and steric tunability to control chain-transfer pathways.

Guided by these insights, we designed a series of unsymmetrical 2,3-bis(arylimino)butane-nickel precatalysts (**C**, Scheme 1) to integrate three critical features, including the 2,3-bis(arylimino)butane backbone, to maximize *M*_w_ and tunable dispersity, the *ortho*-bis(benzhydryl) groups for thermal robustness, as well as a *para*-methyl electron-donating substituent to optimize branching density and elasticity. The newly developed complexes were thoroughly characterized, and their ethylene polymerization behaviors were systematically investigated under varied reaction conditions. Furthermore, the mechanical and elastic properties of the resultant polyethylenes were rigorously analyzed to establish structure-property relationships, particularly how molecular weight, branching density, and crystallinity synergistically govern tensile strength and strain recovery.

## 2. Results and Discussion

### 2.1. Synthesis and Characterization of Ligands and Their Nickel Complexes

**Scheme 2:**
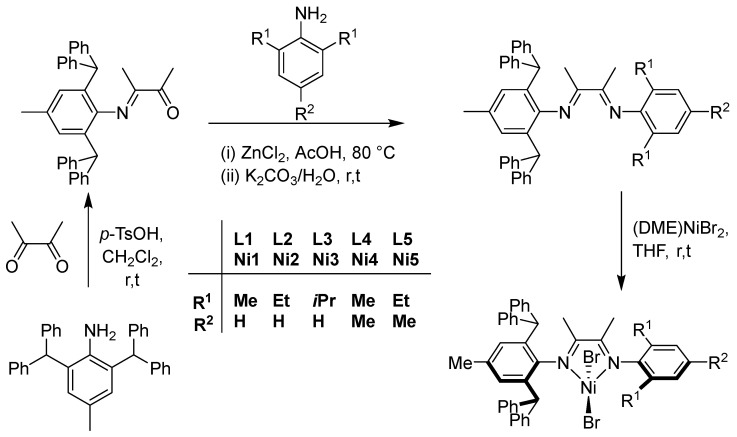
Synthesis of ligands **L1**–**L5** and their nickel complexes **Ni1**–**Ni5**.

The 2,3-bis(arylimino)butane derivatives, {2,6-[CH(C_6_H_5_)_2_]_2_-4-MeC_6_H_2_}N = C(Me)C(Me) = NAr (**L1**–**L5**), where Ar = 2,6-Me_2_C_6_H_3_ (**L1**), Ar = 2,6-Et_2_C_6_H_3_ (**L2**), Ar = 2,6-iPr_2_C_6_H_3_ (**L3**), Ar = 2,4,6-Me_3_C_6_H_2_ (**L4**), Ar = 2,6-Et_2_-4-MeC_6_H_2_ (**L5**), were synthesized via a two-step method, as shown in Scheme 2. The imine-ketone intermediate was initially prepared via a *p*-toluenesulfonic acid-catalyzed condensation of 2,3-butanedione with 2,6-dibenzhydryl-4-methylaniline in dichloromethane under mild conditions, achieving a 65% yield after recrystallization from methanol. A ZnCl_2_-templated strategy was used to perform the second condensation with other anilines. Specifically, the imine-ketone, aniline, and zinc chloride were heated in acetic acid (99.5 w%) at 80 °C for 4 h, followed by demetallation with aqueous K_2_CO_3_ to afford the target ligands **L1**–**L5** in moderate yields (42–57%). The ligands were rigorously characterized by FT-IR, ^1^H/^13^C NMR spectroscopy (see Figures S1–S6), and elemental analysis, confirming their structural integrity and purity. Subsequently, the nickel complexes (**Ni1**–**Ni5**) were synthesized in high yields (>70%) by reacting their respective ligands with (DME)NiBr_2_ in tetrahydrofuran (Scheme 2). Key spectroscopic evidence for nickel complex formation included the redshift of the imine C=N stretching vibration in FT-IR from 1640 cm^−1^ (free ligand) to 1634 cm^−1^ (coordinated to Ni), consistent with successful metal-ligand coordination. Elemental analysis further validated the purity of all new nickel complexes. Moreover, the molecular structures of **Ni4** and **Ni5** were confirmed by single-crystal X-ray diffraction analysis (see Table S1: Crystal data and structure refinement for **Ni4** and **Ni5**), providing atomic-level insights into their geometric configurations.

**Figure 1:**
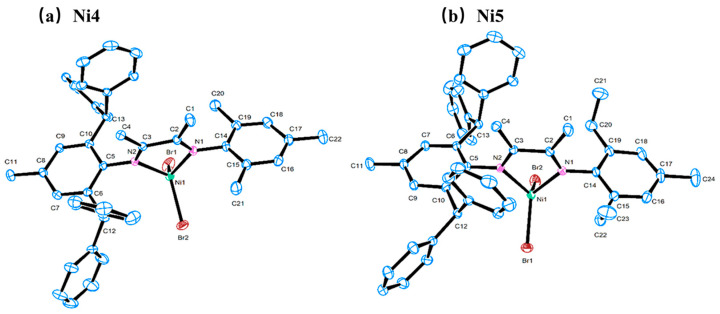
(**a**) ORTEP drawing of **Ni4** and (**b**) **Ni5**. Thermal ellipsoids are shown at the 30% probability level, while the hydrogen atoms have been omitted for clarity.

X-ray-quality crystals of **Ni4** and **Ni5** were grown via controlled diffusion of diethyl ether into their saturated dichloromethane solutions of the complexes (Figure 1a,b). Both complexes adopt a distorted tetrahedral geometry with a four-coordinate nickel center, as evidenced by bond angles and lengths deviating from ideal tetrahedral symmetry. Notably, the *N*-bound phenyl rings—one substituted with sterically demanding dibenzhydryl groups and the other with an alkyl group—display significant tilting relative to the coordination plane [C(2)–C(3)–N(2)–Ni(1)–N(1)]. The dihedral angles in **Ni4** (87.3° and 79.6°) and **Ni5** (89.9° and 85.9°) highlight the steric influence of *ortho-*dibenzhydryl groups. Intriguingly, **Ni5** exhibits angles approaching 90°, suggesting enhanced axial site protection compared to literature-reported symmetrical analogues [52], a feature critical for suppressing catalyst deactivation. Furthermore, the N1–Ni1–N2 and Br1–Ni1–Br2 bond angles in these complexes are slightly smaller than those observed in our previously reported unsymmetrical bis(imino)acenaphthene nickel complexes [53]. As summarized in Table 1, the Ni–N2 bond lengths are slightly longer than the Ni–N1 bond lengths in both **Ni4** and **Ni5**, attributed to steric repulsion from the benzhydryl groups weakening N_imine_ coordination to the nickel center—a trend consistent with our previous findings [52].

### 2.2. Ethylene Polymerization Investigation

To systematically evaluate the catalytic performance of the prepared nickel complexes, we first conducted an initial screening of alkylaluminum cocatalysts using **Ni1** as a model catalyst. Guided by these results, a comprehensive optimization of polymerization parameters—including Al/Ni molar ratio, ethylene pressure, temperature, and reaction duration—was performed to maximize activity and control polymer properties across the entire catalyst series (**Ni1**–**Ni5**).

#### 2.2.1. Optimization of Alkyl Aluminum Cocatalyst Selection

Alkyl aluminum cocatalysts play a multifunctional role in polymerization systems by scavenging impurities to prevent catalyst deactivation, alkylating the nickel center to generate active species, and modulating chain transfer processes to control polymer properties [31,32,33]. Therefore, the catalytic performance of **Ni1** was systematically evaluated using four structurally distinct alkylaluminum cocatalysts: MAO, MMAO, Et_2_AlCl, and EASC. Under identical conditions (Table 2), Et_2_AlCl emerged as the optimal cocatalyst, delivering slightly higher activity (4.61 × 10^6^ g PE mol^−1^ (Ni) h^−1^) than MAO, while exhibiting 9.8-fold higher activity than EASC and 1.64-fold greater efficiency than MMAO. Meanwhile, Ni/Et_2_AlCl shows superior performance by delivering polyethylenes with the highest molecular weight (5.96 × 10^5^ g mol^−1^) and the lowest melting temperature (49.01 °C), attributed to increased branching density from enhanced chain-walking behavior [42]. Notably, despite requiring only 400 equivalents (vs. 2000 for MAO/MMAO), Et_2_AlCl outperformed all tested cocatalysts in both activity and polymer microstructure control. These stark contrasts underscore how cocatalyst steric bulk, Lewis acidity, and activation efficiency critically govern nickel center reactivity and chain-transfer kinetics [54]. Based on these findings, Et_2_AlCl was selected for subsequent optimization studies.

#### 2.2.2. Optimization of Polymerization Conditions Using Ni1 with Et_2_AlCl as Cocatalyst

The **Ni1**/Et_2_AlCl system was systematically optimized to establish optimal conditions for ligand screening. Polymerization tests were conducted across a range of Et_2_AlCl, temperatures, reaction times, and ethylene pressures, with detailed results summarized in Table 3.

**Figure 2:**
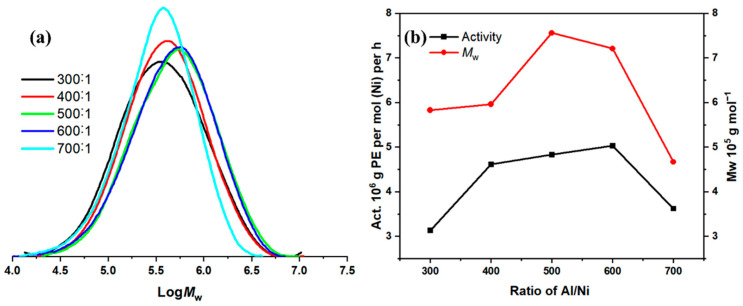
(**a**) GPC curves and (**b**) plots of catalytic activity and molecular weight of the polyethylene produced using **Ni1**/Et_2_AlCl at different Al/Ni molar ratios (entries 1–5, Table 3).

To probe the impact of Et_2_AlCl concentration, polymerization reactions were conducted at Al/Ni molar ratios (300–700) (entries 1–5, Table 3). As shown in Figure 2b, catalytic activity peaked at 5.03 × 10^6^ g PE mol^−1^ (Ni) h^−1^ at an Al/Ni ratio of 600, beyond which excessive cocatalyst accelerated chain transfer to aluminum, reducing both activity and polymer molecular weight. This trend reflects a balance between active site generation (maximized at Al/Ni = 600) and chain transfer overactivation (dominant at Al/Ni = 700) [37]. Similarly, polymer molecular weight (*M*_w_) initially increased, reaching a maximum of 7.56 × 10^5^ g mol^−1^ at Al/Ni = 500 (entries 1–3, Table 3), before declining to 4.67 × 10^5^ g mol^−1^ at Al/Ni = 700 (entries 4–5, Table 3) (Figure 2a). This reduction, attributed to premature chain termination at high cocatalyst levels [42], was accompanied by a decrease in melting temperature (*T*_m_) from 60.75 °C to 40.05 °C, consistent with reduced *M*_w_ and/or increased branching density [45]. These findings align with prior studies [40,41,42,43], underscoring the critical role of cocatalyst concentration in tuning both catalytic performance and polymer microstructure.

**Figure 3:**
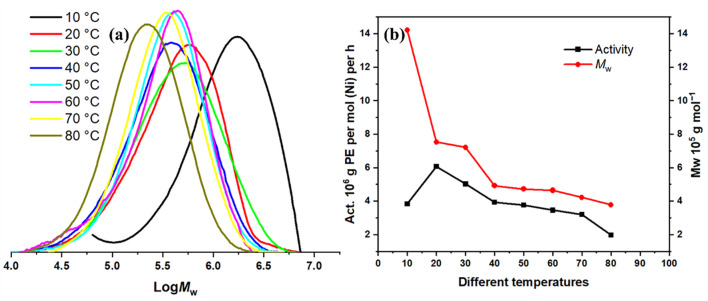
(**a**) GPC traces and (**b**) plots of catalytic activity and molecular weight of the polyethylene produced using **Ni1**/Et_2_AlCl at different run temperatures (entries 4 and 6–12, Table 3).

The reaction temperature serves as a critical parameter governing catalyst stability, polymerization activity, and the molecular weight/structural characteristics of polyethylene (PE) in ethylene polymerization. To systematically evaluate these thermal effects, a series of polymerizations were performed under identical conditions [Al/Ni = 600, ethylene 10 atm] across a temperature gradient of 10–80 °C (entries 4, 6–12, Table 3). As illustrated in Figure 3b, catalytic activity exhibited a distinct volcano-shaped profile: initial activity measured 3.84 × 10^6^ g PE mol^−1^ (Ni) h^−1^ at 10 °C, nearly doubling to 6.07 × 10^6^ g PE mol^−1^ (Ni) h^−1^ at 20 °C. This marked enhancement indicates that temperatures exceeding 10 °C were a prerequisite for complete precatalyst activation. Subsequent temperature elevation beyond 20 °C progressively diminished activity, attributable to thermal decomposition of active nickel species and reduced solubility of ethylene in the solvent [39,40,41]. This temperature-dependent behavior aligns with observations in analogous catalyst systems featuring benzhydryl-substituted unsymmetrical α-diimine ligands [37,38,39,40,41,42,43]. Notably, the polymer molecular weight displayed an inverse correlation with reaction temperature, with ultra-high molecular weight PE (*M*_w_ = 14.22 × 10^5^ g mol^−1^) obtained at 10 °C progressively decreasing to 3.78 × 10^5^ g mol^−1^ at 80 °C (Figure 3b). The molecular weight reduction stems from accelerated chain transfer processes—primarily β-hydride elimination and aluminum-mediated chain transfer—coupled with thermal destabilization of nickel active centers that curtails chain propagation kinetics [25]. Meanwhile, narrow and unimodal molecular weight distributions across all temperatures (Figure 3a) confirmed preservation of single-site catalytic characteristics.

Remarkably, the current catalytic system substituted by a *para*-methyl group maintained substantial activity (1.97 × 10^6^ g PE mol^−1^ (Ni) h^−1^) even at 80 °C, representing a significant enhancement over structurally analogous precatalyst **B** (Scheme 1) [52]. The *para*-methyl substituent enhanced thermal stability through electron-donating effects, while its non-polar nature improved precatalyst solubility in toluene, facilitating efficient cocatalyst activation [37]. The polyethylene obtained at 10 °C displayed a melting temperature of 88.99 °C, progressively decreasing to 15.47 °C at 50 °C. Polymers produced above 50 °C (entries 10–12, Table 3) showed no discernible melting endotherm, indicative of fully amorphous microstructures (discussed in subsequent sections). This contrasts sharply with precatalyst **B**-derived PE obtained at 60 °C [52], which exhibited an obvious melting absorption peak in its DSC curves, thereby validating our previous findings on crystallization modulation through the modification of benzhydryl-methylphenyl ligand architectures [42].

**Figure 4:**
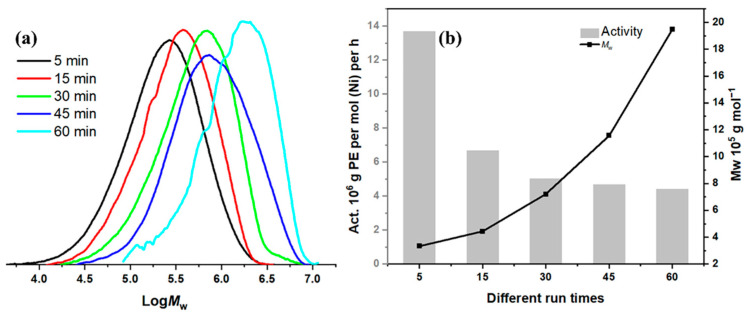
(**a**) GPC traces and (**b**) plots of catalytic activity and molecular weight of the polyethylene produced using **Ni1**/Et_2_AlCl at different run times (entries 4 and 13–16, Table 3).

Subsequently, the **Ni1**/Et_2_AlCl system was subjected to temporal polymerization studies under standardized conditions (Al/Ni = 600, temperature = 20 °C, ethylene = 10 atm). As summarized in Table 3 (entries 7, 13–16), polymerization durations spanning 5–60 min revealed a distinct time-dependent activity profile. The peak activity of 13.67 × 10^6^ g PE mol^−1^ (Ni) h^−1^ was achieved within 5 min, indicative of rapid formation of active species. Activity gradually declined to 4.42 × 10^6^ g PE mol^−1^ (Ni) h^−1^ at 60 min, likely attributable to cumulative catalyst deactivation and mass transfer limitations imposed by polymer accumulation [52]. Meanwhile, the molecular weight of polyethylene exhibited a monotonic increase from 3.33 × 10^5^ g mol^−1^ to 19.47 × 10^5^ g mol^−1^ with extended reaction times (Figure 4b). Notably, ultra-high-molecular-weight polyethylenes were obtained beyond 45 min. The GPC profiles (Figure 4a) displayed a unimodal distribution with narrow dispersity across all time points, confirming sustained single-site catalytic behavior [39]. The persistent high activity up to 4.42 × 10^6^ g PE mol^−1^ (Ni) h^−1^ after 1 h, coupled with molecular weight escalation (19.47 × 10^5^ g mol^−1^), suggests remarkable catalyst longevity (Figure 4b), a phenomenon previously documented in analogous nickel-mediated ethylene polymerization systems [38].

Systematic variation of ethylene pressure (1–10 atm) revealed profound pressure-dependent modulation of catalytic efficiency and polymer characteristics (entries 7, 17–18, Table 3). At atmospheric pressure (1 atm), minimal activity of 0.31 × 10^6^ g PE mol^−1^ (Ni) h^−1^ and low molecular weight PE (*M*_w_ = 3.78 × 10^5^ g mol^−1^) were observed. Elevating the pressure to 5 atm induced a 4.6-fold activity enhancement (1.43 × 10^6^ g PE mol^−1^ (Ni) h^−1^) and 54% molecular weight increase (*M*_w_ = 5.81 × 10^5^ g mol^−1^). Further pressure escalation to 10 atm amplified these effects, achieving 19.6-fold improvement in activity (6.07 × 10^6^ g PE mol^−1^ (Ni) h^−1^) and doubled the molecular weight to 7.53 × 10^5^ g mol^−1^ compared to 1 atm. This pressure-response correlation aligns with established chain propagation kinetics: elevated ethylene pressure increases local monomer concentration at catalytic sites, favoring chain growth over termination pathways (β-H elimination, chain transfer) [39]. Thermal analysis corroborated micro-structural variations, with polyethylenes synthesized at 10 atm exhibiting a higher melting temperature (*T*_m_ = 81.58 °C), indicative of reduced branching degree and enhanced crystallinity. Conversely, atmospheric-pressure polyethylenes displayed a depressed melting temperature (*T*_m_ = 51.50 °C), characteristic of highly branched and low molecular weight amorphous domains.

#### 2.2.3. Ligand Structure Screening Under Optimal Conditions

**Figure 5:**
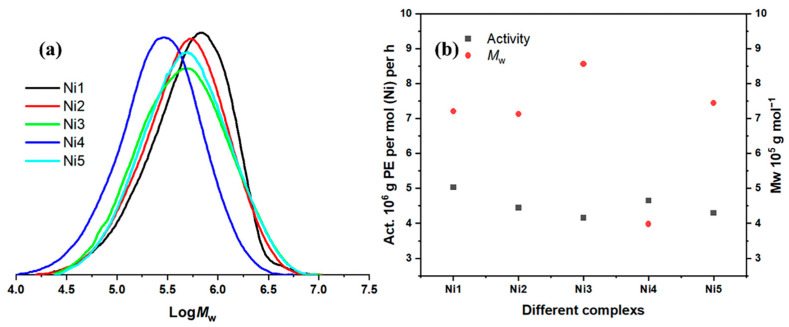
(**a**) GPC traces and (**b**) plots of the catalytic activity and molecular weight of the polyethylene produced using **Ni1**–**Ni5** in combination with Et_2_AlCl (entries 7 and 19–22, Table 3).

Following optimization of **Ni1**/Et_2_AlCl, a systematic evaluation of structural analogs (**Ni2**–**Ni5**) was conducted under identical polymerization conditions (Al/Ni = 600, 20 °C, 10 atm ethylene) to elucidate ligand structure-performance correlations (entries 7, 19–22, Table 3). All catalysts demonstrated high activities (4.16–6.07 × 10^6^ g PE mol^−1^ (Ni) h^−1^) while producing polyethylenes with high molecular weights (3.98–8.56 × 10^5^ g mol^−1^) and narrow dispersities (*M*_w_/*M*_n_ = 2.29–2.50) (Figure 5a). The ligand architectures uniformly incorporated a sterically demanding *N*-2,6-dibenzhydryl-4-methylphenyl group, while systematically varying substituents on the secondary N-aryl moiety. A distinct structure-activity correlation emerged with the activity decreasing in the following order: **Ni1** [2,6-di(Me)] > **Ni4** [2,4,6-tri(Me)] > **Ni2** [2,6-di(Et)] > **Ni5** [2,6-di(Et)-4-Me] > **Ni3** [2,6-di(i-Pr)]. This progression highlights the steric modulation—less hindered analogs (**Ni1** and **Ni4**) exhibited 21% and 8% greater catalytic activity than their bulkier counterparts (**Ni2** and **Ni5,** respectively). The axial alkyl substituents’ steric bulk impedes ethylene coordination and insertion at the active center through spatial occupation of the metal coordination sphere [33]. The most sterically encumbered **Ni3** accordingly exhibited the lowest activities.

Polymer molecular weights were found to inversely correlate with catalytic activity: **Ni3** [2,6-di(i-Pr)] > **Ni1** [2,6-di(Me)] ≈ **Ni5** [2,6-di(Et)-4-Me] > **Ni2** [2,6-di(Et)] > **Ni4** [2,4,6-tri(Me)] (Figure 5a). **Ni3** produced polyethylene with a molecular weight of 8.56 × 10^5^ g mol^−1^, representing 20% and 14% enhancements over **Ni2** and **Ni1**, respectively (entries 7, 19–20, Table 3). This anti-correlation demonstrates steric control over chain propagation kinetics—bulkier ligands suppress chain transfer pathways, extending polymer growth duration. Comparative analysis with benchmark catalyst **B** revealed fundamental distinctions: all current complexes produced unimodal polyethylenes (PDI < 2.5), contrasting sharply with **B**’s bimodal distributions [52]. This dichotomy originates from substituent electronic characteristics—the electron-withdrawing chlorine in **B** facilitates multiple active species formation, while the methyl group’s electron-donating nature in **Ni1**–**Ni5** stabilizes a single catalytically active conformation. The resultant unimodal distributions signify superior control over polymer architecture, particularly advantageous for high-performance polyethylene synthesis [37].

### 2.3. Microstructure Analysis of Polyethylenes

**Figure 6:**
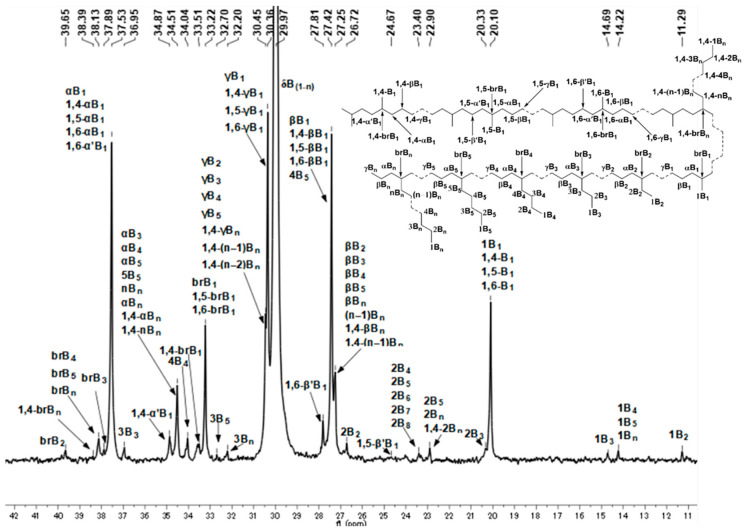
^13^C NMR spectrum of polyethylene sample named PE-Et_2_AlCl-20 obtained at 20 °C using **Ni1**/Et_2_AlCl (entry 7, Table 3).

**Figure 7:**
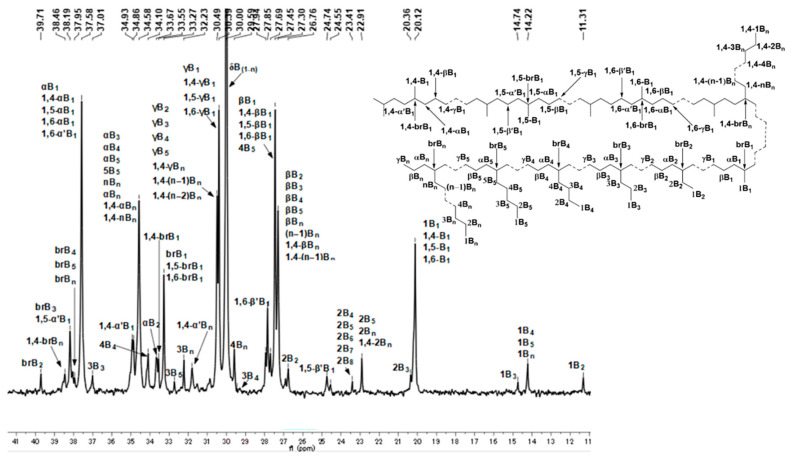
^13^C NMR spectrum of polyethylene sample named PE-Et_2_AlCl-70 obtained at 70 °C using **Ni1**/Et_2_AlCl (entry 11, Table 3).

The melt temperatures of polyethylene are dependent on the reaction conditions and vary significantly with an increase in reaction temperature (entries 4, 6–12, Table 3). Polyethylene produced at 10 °C had a high melt temperature of 88.99 °C, while that produced at 50 °C showed a much lower melt temperature of 15.47 °C. Above 50 °C, no distinct melt temperature was observed, as the polymer transitioned from 35.78% crystallinity at 10 °C to only 8.19% at 50 °C, ultimately becoming fully amorphous above 50 °C. This change in crystallinity is directly related to the branching degree and content [55]. To investigate the branching content of the polyethylenes, high-temperature ^13^C NMR spectroscopy was employed for polyethylene produced at different temperatures. In particular, two polyethylene samples prepared using Ni1/Et_2_AlCl at 20 °C and 70 °C (entries 7 and 11, Table 3), labeled as PE-Et_2_AlCl-20 and PE-Et_2_AlCl-70, were characterized (Figure 6 and Figure 7). The signals were assigned based on previous reports [56]. PE-Et_2_AlCl-20 contained 84 branches/1000 carbons (Table 4), with the predominant types being methyl (84.8%), ethyl (3.1%), propyl (4.0%), butyl (4.9%), amyl (0.8%), and longer chains (2.4%). In comparison, PE-Et_2_AlCl-70 (entry 11, Table 3) exhibited an extremely high level of branching (217 branches per 1000 carbons). The main types of branches were methyl (71.4%), ethyl (5.3%), propyl (6.1%), butyl (8.8%), amyl (2.0%), and longer chains (6.4%). This occurs due to the enhanced chain walking reactions that are more prominent at higher temperatures. At elevated temperatures, the energy barriers for the formation of β-agostic alkyl metal complexes decrease, leading to more frequent chain walking events. Consequently, the polyethylene produced under these conditions exhibits an increased branching degree, which results in a lower melt temperature [37,43]. Compared to previously reported precatalysts **B** (Scheme 1), the replacement of para-Cl with a para-Me group in the prepared nickel complexes increased both the branching degree (**B**: 102 branches/1000C, this work: 217 branches/1000C) and the molecular weight of the polyethylenes [52].

### 2.4. Evaluation of Mechanical Performance in Polyethylenes

**Figure 8:**
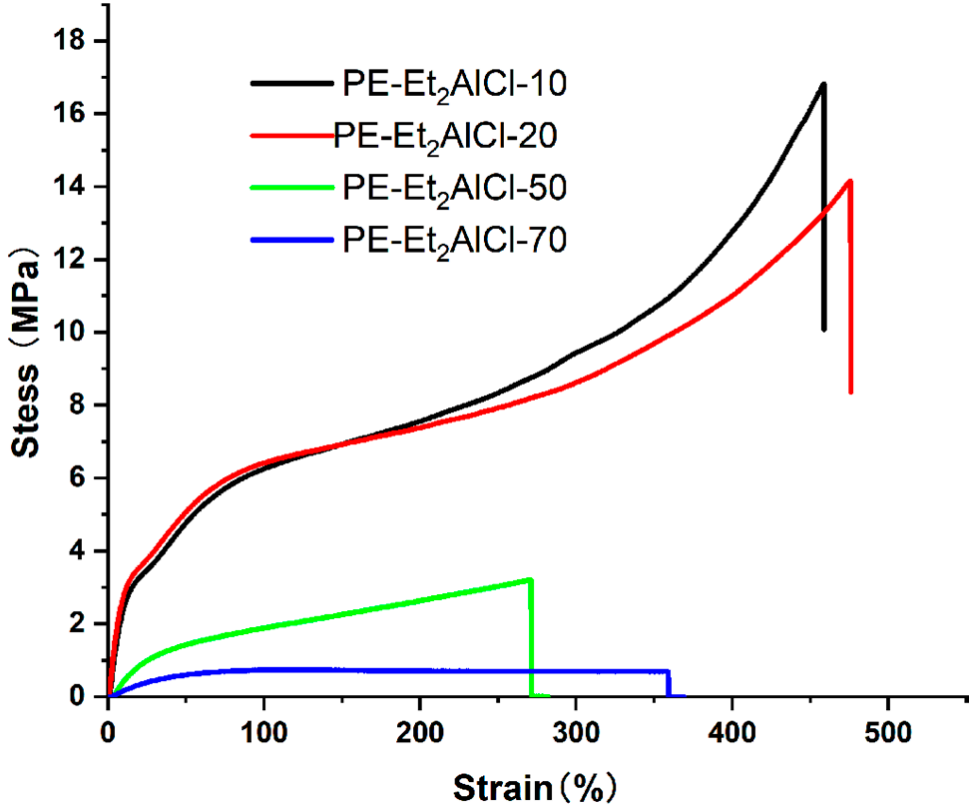
Stress–strain curves for PE-Et_2_AlCl-10, PE-Et_2_AlCl-20, PE-Et_2_AlCl-50, and PE-Et_2_AlCl-70.

The physical properties of polyethylene, such as elasticity, mechanical strength, and toughness, are important for determining its suitability in various applications such as packaging, automotive, and construction. These properties influence the material performance, durability, and resistance to wear and tear in real-world conditions. To assess the physical properties of the prepared polyethylenes, four samples produced at different reaction temperatures (10 °C, 20 °C, 50 °C, 70 °C) using **Ni1**/Et_2_AlCl were selected and named PE-Et_2_AlCl-10 (entry 6, Table 3), PE-Et_2_AlCl-20 (entry 7, Table 3), PE-Et_2_AlCl-50 (entry 9, Table 3), and PE-Et_2_AlCl-70 (entry 11, Table 3). Each sample was tested for tensile stress-strain using a universal tester and for stress-strain recovery via dynamic mechanical analysis (DMA). The complete set of results are provided in Table 5. First, monotonic tensile stress-strain measurements were conducted at room temperature, with each test involving three specimens to ensure consistent results. The stress-strain curves are shown in Figure 8. The polyethylenes PE-Et_2_AlCl-10 and PE-Et_2_AlCl-20 prepared at lower temperatures (10 °C and 20 °C) exhibit higher tensile strength (16.83 MPa and 14.16 MPa) and strain-at-break values (458% and 475%) than previously reported polyethylenes in the literature [57]. This is likely due to the combination of high molecular weight (14.22 × 10^5^ g mol^−1^ and 7.53 × 10^5^ g mol^−1^) and low branching degree (PE-Et_2_AlCl-20, 84 branches/1000C), which in turn is associated with high crystallinity (35.78% and 25.76%). As shown in Table 5, the polyethylene sample PE-Et_2_AlCl-50, prepared at 50 °C, exhibited slightly poorer mechanical properties (*σ*_b_ = 3.21 MPa, *ε*_b_ = 271%) than PE-Et_2_AlCl-10 and PE-Et_2_AlCl-20, likely due to a combination of lower molecular weight (4.72 × 10^5^ g mol^−1^), higher branching, and lower *T*_m_ and *X*_c_ (Table 5). The lowest ultimate tensile stress of 0.74 MPa was observed for PE-Et_2_AlCl-70, likely due to the material being almost amorphous at the run temperature [33,37]. However, it exhibited higher strain compared to PE-Et_2_AlCl-50, possibly because the higher branching at 70 °C results in a softer polymer, leading to slightly higher strain-at-break values (358%) compared to the sample prepared at 50 °C (271%).

**Figure 9:**
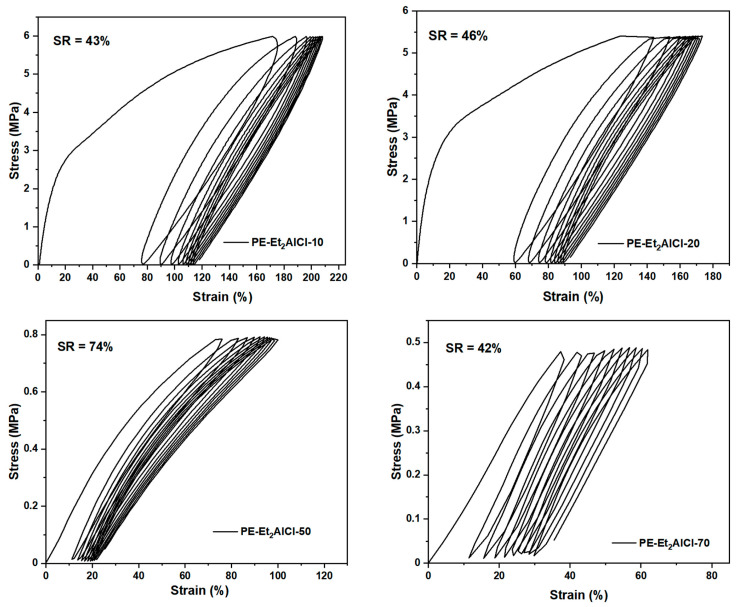
Stress–strain recovery tests for PE-Et_2_AlCl-10, PE-Et_2_AlCl-20, PE-Et_2_AlCl-50, and PE-Et_2_AlCl-70 at 30 °C.

Figure 9 and Table 5 show the results of stress–strain recovery tests using DMA. These tests were conducted at 30 °C, with each cycle repeated up to ten times. The stress-strain hysteresis loops exhibited consistent recovery levels for all samples after the first cycle, indicating that the polyethylenes displayed characteristics of thermoplastic elastomers (TPEs). Despite its high *M*_w_ (4.22 × 10^5^ g mol^–1^), the high branching density (217 branches/1000C) of PE-Et_2_AlCl-70 resulted in only 42% elastomeric recovery under a tensile stress of 0.5 MPa. The absence of a crystalline region and its completely amorphous nature rendered the polymer soft, with lower mechanical strength and reduced elastic recovery. The PE-Et_2_AlCl-50 sample demonstrated superior strain recovery (74%), attributed to its balanced branching and moderate crystallinity (8.19%). In contrast, PE-Et_2_AlCl-10 and PE-Et_2_AlCl-20 achieved recoveries of 43% and 46%, respectively, likely due to their greater crystalline content. These results indicate that the branching degree and crystallinity of polyethylene significantly influence its mechanical and elastic properties. Excessive crystallinity reduces elasticity, while a completely amorphous nature results in a soft polymer with limited elastic recovery [50,52].

## 3. Materials and Methods

### 3.1. Synthesis of Monoketone and Ligands (**L1**–**L5**)

**2-(2,6-Dibenzhydryl-4-methylphenylimino)butanone**

A solution of 2,6-diphenylmethyl-4-methylaniline (8.79 g, 20.0 mmol), 2,3-butanedione (1.72 g, 20.0 mmol), and a catalytic amount of p-toluenesulfonic acid (0.879 g) in dichloromethane (300 mL) was stirred at ambient temperature for 4 h. Afterward, the solvent was removed under reduced pressure, and the crude product was purified by recrystallization from methanol, affording 6.60 g of yellow solid with 65% yield. ^1^H NMR (400 MHz, CDCl_3_, TMS): δ 7.26–7.14 (m, 16H, Py–*H*), 7.01 (t, *J* = 2.0 Hz, 4H, Py–*H*), 6.64 (s, 2H, Py–*H*), 5.09 (s, 2H, Ar–C*H*(Ph)_2_), 2.31 (s, 3H, O=C–C*H*_3_), 2.15 (s, 3H, Ar–C*H*_3_), 0.68 (s, 3H, N=C–C*H*_3_). ^13^C NMR (100 MHz, CDCl_3_, TMS): δ 199.3 (O=*C*–CH_3_), 168.5 (N=*C*–CH_3_), 144.3, 142.5, 142.0, 132.3, 130.8, 129.4, 129.1, 128.3, 127.0, 126.2, 126.0, 52.1, 24.7, 21.1, 14.3. FT-IR (cm^–1^): 3059 (w), 3026 (w), 2921 (w), 1702 (s), 1649 (m), 1599 (w), 1572 (w), 1494 (m), 1439 (m), 1356 (m), 1262 (m), 1183 (m), 1119 (m), 1077 (m) 1031(m), 924 (w), 892 (w), 768 (m), 742 (m), 697 (s), 684 (m). Anal. calcd for C_37_H_33_NO (507.68): C, 87.54; H, 6.55; N, 2.79%. Found: C, 87.29; H, 6.59; N, 2.73%.

**2-(2,6-Dibenzhydryl-4-methylphenylimino)-3-(2,6-dimethylphenylimino)butane (L1)**

To a 25 mL round-bottomed flask, equipped with a stir bar, was added zinc(II) chloride (0.20 g, 1.5 mmol), 2-(2,6-dibenzhydryl-4-methylphenylimino)butanone (0.76 g, 1.5 mmol), 2,6-dimethylaniline (0.18 g, 1.5 mmol), and acetic acid (1 mL). The reaction mixture was stirred and heated for 4 h at 80 °C. Once cooled to room temperature, diethyl ether (10 mL) was added and the resulting yellow precipitate was filtered. This intermediate zinc(II) chloride complex was then dissolved in dichloromethane and a saturated aqueous solution of potassium carbonate (K_2_CO_3_) was added and the stirred at room temperature for 1.5 h [58,59,60]. Using a separating funnel, the organic layer was extracted and washed with water three times and dried over anhydrous magnesium sulfate (MgSO_4_). After removing the volatiles by rotary evaporation, the product was recrystallized from hexane to yield **L1** as a yellow powder (0.52 g, 57%). ^1^H NMR (400 MHz, CDCl_3_, TMS): δ 7.27–7.22 (m, 8H, Py–*H*), 7.20–7.15 (m, 4H, Py–*H*), 7.09–7.03 (m, 10H, Py–*H*), 6.91 (t, *J* = 8.0 Hz, 1H, Py–*H*), 6.65 (s, 2H, Py–*H*), 5.23 (s, 2H, Ar–C*H*(Ph)_2_), 2.17 (s, 3H, Ar–C*H*_3_), 1.81 (s, 3H, N=C–C*H*_3_), 1.09 (s, 6H, Py–C*H*_3_), 0.85 (s, 3H, N=C–C*H*_3_). ^13^C NMR (100 MHz, CDCl_3_, TMS): δ 170.0 (N=*C*–CH_3_), 167.8 (N=*C*–CH_3_), 148.6, 145.8, 143.7, 142.6, 131.9, 131.6, 129.9, 129.6, 128.8, 128.6, 128.2, 128.0, 126.5, 126.2, 124.6, 123.2, 52.5, 21.5, 18.1, 16.1, 16.0. FT-IR (cm^–1^): 3024 (w, *v_Py–H_*), 2968 (w, *v*_N=C*–C–H*_), 1635 (m, *v_C=N_*), 1597 (w, *v_Py_*), 1493 (m, *v_Py_*), 1447 (m, *v_Py_*), 1419 (m, *v_Py_*), 1351 (m, *v_Py_*), 1257 (m, *v_C–N_*), 1199 (m, *v_C–N_*), 1122 (m, *v_Py_*), 1081 (m, *v_Py_*), 1028 (m, *v_Py_*), 924 (w, *v_Py_* ), 915 (w, *v_Py_*), 885 (m, *v_Py_*), 859 (m, *v*_Ar–*C–H*_), 803 (s, *v*_Ar–*C–H*_), 746 (m, *v*_Ar-*C-H*_), 698 (s, *v*_Ar–CH(*Ph-H*)_). Anal. calcd for C_45_H_42_N_2_ (610.85): C, 88.48; H, 6.93; N, 4.59%. Found: C, 88.33; H, 6.82; N, 4.66%.

**2-(2,6-Dibenzhydryl-4-methylphenylimino)-3-(2,6-diethylphenylimino)butane (L2)**

Using a similar procedure as described for **L1** but using 2,6-diethylaniline as the aniline gave **L2** as a yellow solid (0.48 g, 50%). ^1^H NMR (400 MHz, CDCl_3_, TMS): δ 7.28–7.17 (m, 11H, Py–*H*), 7.11–6.99 (m, 12H, Py–*H*), 6.67 (s, 2H, Py–*H*), 5.25 (s, 2H, Ar–C*H*(Ph)_2_), 2.31 (t, *J* = 8.0 Hz, 4H, Py–C*H*_2_CH_3_), 2.18 (s, 3H, Ar–C*H*_3_), 1.84 (s, 3H, N=C–C*H*_3_), 1.18 (s, *J* = 8.0 Hz, 6H, Py–CH_2_C*H*_3_), 0.89 (s, 3H, N=C–C*H*_3_). ^13^C NMR (100 MHz, CDCl_3_, TMS): δ 170.0 (N=*C*–CH_3_), 167.7 (N=*C*–CH_3_), 147.5, 145.7, 143.8, 142.6, 131.9, 131.6, 130.4, 129.9, 129.6, 128.9, 128.6, 128.2, 126.5, 126.2, 126.0, 123.6, 52.4, 24.6, 21.5, 16.4, 16.1, 14.0. FT-IR (cm^–1^): 3024 (w), 2963 (w), 1640 (m, *v*_C=N_), 1597 (m), 1493 (m), 1446 (m), 1419 (m), 1356 (m), 1255 (m), 1194 (m), 1121 (m), 1076 (m), 1029 (m), 1008 (w), 913 (w), 859 (m), 801 (m), 748 (m), 697 (s). Anal. calcd for C_47_H_46_N_2_ (638.90): C, 88.36; H, 7.26; N, 4.38%. Found: C, 88.10; H, 7.38; N, 4.31%.

**2-(2,6-Dibenzhydryl-4-methylphenylimino)-3-(2,6-diisopropylphenylimino)butane (L3)**

Using a similar procedure as described for **L1** but using 2,6-diisopropylaniline as the aniline gave **L3** as a yellow solid (0.42 g, 42%). ^1^H NMR (400 MHz, CDCl_3_, TMS): δ 7.28–7.24 (m, 8H, Py–*H*), 7.20–7.17 (m, 4H, Py–*H*), 7.15–7.09 (m, 6H, Py–*H*), 7.08–7.04 (m, 5H, Py–*H*), 6.66 (s, 2H, Py–*H*), 5.25 (s, 2H, Ar–C*H*(Ph)_2_), 2.61 (m, 2H, Py–C*H*(CH_3_)_2_), 2.18 (s, 3H, Ar–C*H*_3_), 1.85 (s, 3H, N=C–C*H*_3_), 1.22 (d, *J* = 6.8 Hz, 6H, Py–CH(C*H*_3_)_2_), 1.17 (d, *J* = 6.8 Hz, 6H, Py–CH(C*H*_3_)_2_), 0.88 (s, 3H, N=C–C*H*_3_). ^13^C NMR (100 MHz, CDCl_3_, TMS): δ 170.0 (N=*C*–CH_3_), 168.0 (N=*C*–CH_3_), 146.2, 145.8, 143.9, 142.7, 135.1, 131.9, 131.6, 129.9, 129.6, 128.9, 128.6, 128.2, 126.5, 126.2, 123.9, 123.1, 52.4, 28.3, 23.5, 23.3, 21.5, 16.8, 16.4. FT-IR (cm^–1^): 3025 (w), 2961 (w), 2921 (w), 1641 (m, *v*_C=N_), 1597 (w), 1597 (m), 1493 (m), 1443 (m), 1357 (m), 1326 (m), 1250 (m), 1186 (m), 1117 (m), 1075 (m), 1021 (m), 997 (w), 914 (w), 857 (w), 794 (m), 767 (m), 697 (s). Anal. calcd for C_49_H_50_N_2_ (666.95): C, 88.24; H, 7.56; N, 4.20%. Found: C, 88.01; H, 7.70; N, 4.29%.

**2-(2,6-Dibenzhydryl-4-mehtylphenylimino)-3-(2,4,6-trimethylphenylimino)butane (L4)**

Using a similar procedure as described for **L1** but using 2,4,6-trimethylaniline as the aniline gave **L4** as a yellow solid (0.45 g, 48%). ^1^H NMR (400 MHz, CDCl_3_, TMS): δ 7.29–7.24 (m, 8H, Py–*H*), 7.22–7.17 (m, 4H, Py–*H*), 7.12–7.06 (m, 8H, Py–*H*), 6.88 (s, 2H, Py–*H*), 6.68 (s, 2H, Py–*H*), 5.25 (s, 2H, Ar–C*H*(Ph)_2_), 2.29 (s, 3H, Py–C*H*_3_), 2.19 (s, 3H, Ar–C*H*_3_), 1.83 (s, 3H, N=C–C*H*_3_), 1.98 (s, 6H, Py–C*H*_3_), 0.88 (s, 3H, N=C–C*H*_3_). ^13^C NMR (100 MHz, CDCl_3_, TMS): δ 170.1 (N=*C*–CH_3_), 168.0 (N=*C*–CH_3_), 146.2, 145.8, 143.8, 142.6, 132.4, 131.8, 131.6, 129.9, 128.8, 128.6, 128.2, 126.5, 126.2, 124.5, 52.5, 21.5, 20.8, 18.0, 16.2, 15.9. FT-IR (cm^–1^): 3022 (w), 2920 (w), 1634 (m, *v*_C=N_), 1598 (m), 1570 (w), 1492 (m), 1359 (m), 1252 (m), 1201 (w), 1124 (m), 1075 (m), 1029 (m), 913 (w), 854 (m), 768 (w), 741 (m), 696 (s). Anal. calcd for C_46_H_44_N_2_ (624.87): C, 88.42; H, 7.10; N, 4.48%. Found: C, 88.59; H, 7.00; N, 4.42%.

**2-(2,6-Dibenzhydryl-4-mehtylphenylimino)-3-(2,6-dimethyl-4-ethylphenylimino)butane (L5)**

Using a similar procedure as described for **L1** but using 2,6-diethyl-4-methylaniline as the aniline gave **L5** as a yellow solid (0.43 g, 44%). ^1^H NMR (400 MHz, CDCl_3_, TMS): δ 7.28–7.17 (m, 10H, Py–*H*), 7.12–6.98 (m, 10H, Py–*H*), 6.91 (s, 2H, Py–*H*), 6.67 (s, 2H, Py–*H*), 5.26 (s, 2H, Ar–C*H*(Ph)_2_), 2.31 (s, 3H, Py–C*H*_3_), 2.30–2.25 (m, 4H, Py–C*H*_2_CH_3_), 2.19 (s, 3H, Ar–C*H*_3_), 1.84 (s, 3H, N=C–C*H*_3_), 1.17 (t, *J* = 7.6 Hz, 6H, Py–CH_2_C*H*_3_), 0.90 (s, 3H, N=C–C*H*_3_). ^13^C NMR (100 MHz, CDCl_3_, TMS): δ 170.1 (N=*C*–CH_3_), 168.0 (N=*C*–CH_3_), 145.8, 145.1, 143.1, 142.7, 132.7, 131.6, 131.6, 130.3, 129.9, 129.5, 128.2, 126.5, 126.2, 124.5, 52.4, 24.6, 28.8, 21.5, 16.4, 14.3, 14.1. FT-IR (cm^–1^): 3023 (w), 3010 (w), 2959 (w), 1642 (m, *v*_C=N_), 1599 (m), 1572 (w), 1493 (m), 1419 (w), 1359 (m), 1249 (m), 1203 (m), 1124 (m), 1075 (m), 1030 (m), 913 (w), 859 (w), 768 (w), 741 (m), 696 (s). Anal. calcd for C_48_H_48_N_2_ (652.93): C, 88.30; H, 7.41; N, 4.29%. Found: C, 88.11; H, 7.53; N, 4.20%.

### 3.2. Synthesis of Nickel Complexes (**Ni1**–**Ni5**)

**2-(2,6-Dibenzhydryl-4-methylphenylimino)-3-(2,6-dimethylphenylimino)butane-NiBr_2_ (Ni1)**

The complexes **Ni1**–**Ni5** were prepared by the treatment of (DME)NiBr_2_ with the corresponding ligands (**L1**–**L5**) in THF. The preparation of **Ni1** is outlined as follows. **L1** (0.1 g, 0.16 mmol) and (DME)NiBr_2_ (0.05 g, 0.15 mmol) were added to a Schlenk tube, along with 10 mL of dichloromethane. The reaction mixture was stirred for 12 h at room temperature, followed by the addition of absolute diethyl ether (10 mL) to precipitate the complex. The precipitate was washed with diethyl ether and dried under vacuum, yielding a brick-red powder of **Ni1** (70%, 0.093 g). FT-IR (cm^–1^): 3027 (w), 2917 (w), 2923 (w), 2866 (w), 1636 (m, *v*_C=N_), 1599 (m), 1494 (w), 1472 (w), 1441 (m), 1376 (m), 1301 (m), 1219 (m), 1152 (m), 1118 (m), 1075 (m), 1033 (m), 1003 (w), 916 (m), 828 (w), 800 (w), 770 (m), 751 (m), 698 (s). Anal calcd for C_45_H_42_Br_2_N_2_Ni (829.35): C, 65.17; H, 5.10; N, 3.38%. Found C, 64.99; H, 5.22; N, 3.10%.

**2-(2,6-Dibenzhydryl-4-methylphenylimino)-3-(2,6-diethylphenylimino)butane-NiBr_2_ (Ni2)**

A similar approach to that used for **Ni1** was employed, replacing **L1** with **L2** as the ligand. **Ni2** was isolated as a brick red complex (0.099 g, 72%). FT-IR (cm^–1^): 3329 (w), 2969 (w), 2933 (w), 1637 (m, *v*_C=N_), 1602 (m), 1543 (w), 1491 (m), 1445 (m), 1400 (w), 1380 (w), 1340 (w), 1073 (w), 990 (m), 980 (w), 970 (w), 940 (w), 900 (w), 864 (m), 829 (m), 791 (m), 767 (m), 700 (s). Anal calcd for C_47_H_46_Br_2_N_2_Ni (857.40): C, 65.84; H, 5.41; N, 3.27%. Found: C, 65.55; H, 5.20; N, 3.10%.

**2-(2,6-Dibenzhydryl-4-methylphenylimino)-3-(2,6-diisopropylphenylimino)butane-NiBr_2_ (Ni3)**

A similar approach to that used for **Ni1** was employed, replacing **L1** with **L3** as the ligand. **Ni3** was isolated as a brick red complex (0.120 g, 85%). FT-IR (cm^–1^): 3224 (w), 3024 (w), 2960 (m), 2923 (w), 2790 (w), 1634 (m, *v*_C=N_), 1600 (m), 1576 (w), 1494 (m), 1447 (m), 1413 (m), 1378 (s), 1323 (w), 1302 (w), 1252 (w), 1214 (m), 1153 (m), 1123 (m), 1033 (m), 995 (m), 912 (m), 861 (m), 830 (m), 791 (m), 763 (m), 741 (m), 698 (s). Anal calcd for C_49_H_50_Br_2_N_2_Ni (885.45): C, 66.47; H, 5.69; N, 3.16%. Found: C, 66.21; H, 5.82; N, 3.29%.

**2-(2,6-Dibenzhydryl-4-mehtylphenylimino)-3-(2,4,6-trimethylphenylimino)butane-NiBr_2_ (Ni4)**

A similar approach to that used for **Ni1** was employed, replacing **L1** with **L4** as the ligand. **Ni4** was isolated as a brick red complex (0.106 g, 79%). FT-IR (cm^–1^): 3120 (w), 2916 (m), 2762 (w), 1639 (m, *v*_C=N_), 1601 (m), 1491 (m), 1413 (m), 1380 (m), 1361 (m), 1302 (m), 1226 (m), 1167 (m), 1124 (m), 1074 (m), 1036 (m), 997 (w), 974 (w), 912 (w), 859 (m), 828 (m), 800 (m), 753 (m), 710 (s). Anal calcd for C_46_H_44_Br_2_N_2_Ni (843.37): C, 65.51; H, 5.26; N, 3.32%. Found: C, 65.21; H, 5.00; N, 3.11%.

**2-(2,6-Dibenzhydryl-4-mehtylphenylimino)-3-(2,6-dimethyl-4-ethylphenylimino)butane-NiBr_2_ (Ni5)**

A similar approach to that used for **Ni1** was employed, replacing **L1** with **L5** as the ligand. **Ni5** was isolated as a brick red complex (0.100 g, 72%). FT-IR (cm^–1^): 3112 (w), 2960 (m), 2731 (w), 1637 (m, *v*_C=N_), 1574 (m), 1494 (m), 1449 (m), 1392 (m), 1380 (m), 1359 (w), 1330 (w), 1311 (w), 1250 (m), 1219 (m), 1173 (m), 1125 (m), 1032 (m), 1000 (m), 914 (w), 866 (m), 828 (m), 780 (w), 751 (m), 691 (s). Anal calcd for C_47_H_45_Br_2_N_2_Ni (871.43): C, 66.16; H, 5.55; N, 3.21%. Found: C, 66.41; H, 5.10; N, 3.45%.

## 4. Conclusions

In summary, a series of unsymmetrical 2,3-bis(arylimino)butane-nickel precatalysts featuring a sterically hindered N-2,6-bis(benzhydryl)-4-methylphenyl group were successfully prepared and thoroughly characterized. These nickel precatalysts demonstrated exceptional catalytic performance in ethylene polymerization, marked by outstanding thermal stability, high polymer molecular weights, controlled unimodal dispersity, and tunable material properties. Notably, the nickel precatalysts maintained remarkable activity even at an elevated temperature of 80 °C (1.97 × 10^6^ g PE mol^−1^ (Ni) h^−1^) and produced ultra-high molecular weight polyethylene up to 1.95 × 10^6^ g mol^−1^, underscoring the exceptional stability of the active catalytic species. Moreover, the polymerization temperature emerged as a critical parameter for tailoring polymer architecture and performance, with the increase in the reaction temperature inducing a significant rise in branching density (from 84 to 217 branches/1000C), crystallinity, and melting temperature. These microstructural modifications directly governed the mechanical properties of the resulting polyethylenes, achieving a tensile strength of 16.83 MPa and strain recovery values as high as 74%—a hallmark of thermoplastic elastomers (TPEs). The synergy between controlled branching, high molecular weight, and dynamic chain entanglement enabled the polyethylene to exhibit both robust mechanical strength and elastic recovery, positioning it as a promising candidate for advanced TPE applications.

## Figures and Tables

**Table 1:** Selected bond lengths and angles for **Ni4** and **Ni5**.

Nickel Complexes	Ni4	Ni5
Bond length (Å)		
Ni(1)–N(1)	1.999(3)	2.007(2)
Ni(1)–N(2)	2.016(3)	2.020(2)
Ni(1)–Br(1)	2.3063(9)	2.3387(8)
Ni(1)–Br(2)	2.3425(7)	2.3159(9)
N(1)–C(2)	1.286(4)	1.276(4)
N(1)–C(14)	1.448(4)	1.445(4)
N(2)–C(3)	1.289(4)	1.288(4)
N(2)–C(5)	1.450(4)	1.447(3)
Bond angles (°)		
Br(1)–Ni(1)–Br(2)	119.36(3)	119.77(3)
N(1)–Ni(1)–Br(1)	122.20(8)	113.32(7)
N(1)–Ni(1)–Br(2)	101.83(8)	110.88(7)
N(1)–Ni(1)–N(2)	80.84(10)	80.99(10)
N(2)–Ni(1)–Br(1)	117.25(7)	111.20(7)
N(2)–Ni(1)–Br(2)	108.47(7)	114.34(7)

**Table 2:** Ethylene polymerization results obtained with **Ni1** with different cocatalysts ^a^.

Entry	Cocat.	T/°C	t/min	Al/Ni	Act. ^b^	*M*_w_ ^c^	*M*_w_/*M*_n_ ^c^	*T*_m_ ^d^
1	MAO	30	30	2000	4.11	4.23	2.15	85.11
2	MMAO	30	30	2000	2.81	3.52	3.99	66.01
3	EASC	30	30	400	0.47	5.12	2.22	61.61
4	Et_2_AlCl	30	30	400	4.61	5.96	2.35	49.01

^a^ Conditions: cat. **Ni1** (2 μmol) and 100 mL toluene, 10 atm C_2_H_4_. ^b^ 10^6^ g PE mol^−1^ (Ni) h^−1^. ^c^ *M*_w_ 10^5^ g mol^−1^; *M*_w_ and *M*_w_/*M*_n_ determined by GPC. ^d^ Determined by DSC.

**Table 3:** Ethylene polymerization results obtained under different conditions using **Ni1**–**Ni5** in combination with Et_2_AlCl ^a^.

Entry	Precat.	T/°C	t/min	Al/Ni	Activity ^b^	*M*_w_ ^c^	*M*_w_/*M*_n_ ^c^	*T*_m_ ^d^
1	**Ni1**	30	30	300	3.13	5.83	2.67	60.75
2	**Ni1**	30	30	400	4.61	5.96	2.35	49.01
3	**Ni1**	30	30	500	4.83	7.56	2.54	47.40
4	**Ni1**	30	30	600	5.03	7.21	2.60	45.92
5	**Ni1**	30	30	700	3.62	4.67	2.15	45.05
6	**Ni1**	10	30	600	3.84	14.22	2.23	88.99
7	**Ni1**	20	30	600	6.07	7.53	2.37	81.58
8	**Ni1**	40	30	600	3.94	4.92	2.26	41.04
9	**Ni1**	50	30	600	3.77	4.72	1.97	15.47
10	**Ni1**	60	30	600	3.46	4.64	2.22	– ^g^
11	**Ni1**	70	30	600	3.20	4.22	1.85	– ^g^
12	**Ni1**	80	30	600	1.97	3.78	1.95	– ^g^
13	**Ni1**	20	5	600	13.67	3.33	2.48	80.38
14	**Ni1**	20	15	600	6.68	4.44	2.21	54.99
15	**Ni1**	20	45	600	4.67	11.58	2.47	74.42
16	**Ni1**	20	60	600	4.42	19.47	2.15	75.81
17 ^e^	**Ni1**	20	30	600	0.31	3.78	1.95	51.50
18 ^f^	**Ni1**	20	30	600	1.43	5.81	2.31	52.45
19	**Ni2**	20	30	600	4.45	7.13	2.44	53.88
20	**Ni3**	20	30	600	4.16	8.56	2.29	69.80
21	**Ni4**	20	30	600	4.65	3.98	2.35	78.64
22	**Ni5**	20	30	600	4.3	7.44	2.50	76.73

^a^ Conditions: precat. **Ni** (2 μmol), cocat. Et_2_AlCl, 100 mL toluene, 10 atm C_2_H_4_. ^b^ 10^6^ g PE mol^−1^ (Ni) h^−1^. ^c^ *M*_w_ 10^5^ g mol^−1^; *M*_w_ and *M*_w_/*M*_n_ determined by GPC. ^d^ Determined by DSC. ^e^ 1 atm of ethylene. ^f^ 5 atm of ethylene. ^g^ Broad and weak endotherms, amorphous-like polyethylenes.

**Table 4:** Branching degree and content of the polyethylenes produced at 20 and 70 °C using **Ni1**/Et_2_AlCl.

PE Sample	Branches /1000Cs	Branching Composition (%)					
		Me	Et	Pr	Bu	Amyl	Longer Branch
PE-Et_2_AlCl-20	84	84.8	3.1	4.0	4.9	0.8	2.4
PE-Et_2_AlCl-70	217	71.4	5.3	6.1	8.8	2.0	6.4

**Table 5:** Branching degree and composition of the PEs.

PE Sample	T (°C)	*M*_w_ (10^5^) ^a^	*T*_m_ (°C) ^b^	*X*_c_ (%) ^b^	*σ*_b_ (MPa) ^c^	*ε*_b_ (%) ^c^	SR (%) ^d^
PE-Et_2_AlCl-10	10	14.22	88.99	35.78	16.83	458	43
PE-EtAlCl_2_-20	20	7.53	81.58	29.76	14.16	475	46
PE-Et_2_AlCl-50	50	4.72	15.47	8.19	3.21	271	74
PE-Et_2_AlCl-70	70	4.22	– ^e^	–	0.74	358	42

^a^ Determined by GPC, unit: g mol^–1^. ^b^ Determined by DSC; *X*_c_ = Δ*H*_f_ (*T*_m_) = Δ*H*_f_° (*T*_m_°); Δ*H*_f_° (*T*_m_°) = 248.3 J g^–1^. ^c^ Determined by using a universal tester. ^d^ Strain recovery values (SR) were calculated by using the standard formula SR = 100(*ε*_a_ − *ε*_r_)/*ε*_a_, where *ε*_a_ is the applied strain and *ε*_r_ is the strain in the cycle at 0 loads after 10 cycles. ^e^ Broad and weak endotherms, amorphous-like polyethylenes.

## Data Availability

CCDC reference numbers 2431758 and 2431759 contain supplementary crystallographic information for **Ni4** and **Ni5** (see Supplementary Materials), and the data presented in this study are available in this article.

## Supplementary Materials

The following supporting information can be downloaded at: https://www.mdpi.com/article/10.3390/molecules30081847/s1. General Considerations, Table S1: Crystal data and structure refinement for **Ni4** and **Ni5**, Figures S1–S6. ^1^H NMR (400 MPa) spectrum of 2-(2,6-dibenzhydryl-4- methylphenylimino)butanone, **L1**–**L5** (recorded in CDCl_3_ at 25 °C). Refs. [61,62] are cited in the Supplementary Materials.
