# Supplementary material for: Tuning 2,3-Bis(arylimino)butane-nickel Precatalysts for High-Molecular-Weight Polyethylene Elastomers
Source: Molecules. 2025 Apr 20;30(8):1847. doi: 10.3390/molecules30081847 (PMC12029195; doi:10.3390/molecules30081847)
Supplement: Supplementary file 1 [file molecules-30-01847-s001.zip › molecules-3565244-supplementary.pdf]

**Supplementary Information**

**for**

# Tuning 2,3-Bis(arylimino)butane-Nickel Precatalysts for High-Molecular-Weight Polyethylene Elastomers

**Dongzhi Zhu [1,2], Dedong Jia [2], Qiuyue Zhang [2], Yanping Ma [2,*], Qaiser Mahmood [3] and Wen-Hua Sun [2,*]**

[1]Guangxi Key Laboratory of Advanced Structural Materials and Carbon Neutralization, School of Materials and Environment, Guangxi Minzu University, Nanning 530105, China; dzzhu1992@iccas.ac.cn (D.Z.)

[2]Key Laboratory of Engineering Plastics, Beijing National Laboratory for Molecular Sciences, Institute of Chemistry Chinese Academy of Sciences, Beijing 100190, China; jiaded@ahut.edu.cn (D.J.), zhangqiuyue@iccas.ac.cn (Q.Z.)

[3]Chemistry and Chemical Engineering Guangdong Laboratory, Shantou, 515031, China ; qaiser@ccelab.com.cn (Q.M.)

*Correspondence: myanping@iccas.ac.cn (Y.M.), whsun@iccas.ac.cn (W.-H.S.)

**Table of contents**

1 **General considerations**

2 **General Procedure for Ethylene Polymerization**

3 **X-ray Crystallographic Studies**

4 **$^{1}$H NMR spectra of imino-ketone and ligands (Figure S1-S6)**

5 **Reference**

## 1. General Considerations

All procedures involving air- and moisture-sensitive compounds were conducted under a nitrogen atmosphere using standard Schlenk techniques. Before use, toluene was refluxed over sodium and distilled for 24 h under a nitrogen atmosphere. The cocatalyst MAO (1.46 M solution in toluene) and MMAO (2.00 M in n-heptane) were purchased from Anhui Botai Electronic Materials Co. (Chuzhou, China). Diethylaluminum chloride ($Et_2AlCl$, 1.17 M in toluene) and ethylaluminum sesquichloride (EASC, 0.87 M in toluene) were purchased from Yantai Lianli Chemical Co. (Shandong, China). Other reagents were purchased from Aldrich (Shanghai, China), Innochem Technology Co., Ltd. (Beijing, China), or local suppliers. High-purity ethylene was purchased from Beijing Yanshan Petrochemical Company (Beijing, China) and used as received. $^{1}H$ and $^{13}C$ NMR spectra of ligands were recorded on a Bruker DMX 400 MHz instrument (Bruker Corporation, Billerica, MA, USA) at ambient temperature using tetramethylsilane (TMS) as an internal standard. IR spectra of ligands and nickel complexes were recorded on a PerkinElmer System 2000 FT-IR spectrometer (PerkinElmer Scientific, Waltham, MA, USA). Elemental analyses were conducted using a Flash EA 1112 microanalyzer (Thermo Fisher Scientific, Waltham, MA, USA). Molecular weights ($M$w) and molecular weight distributions ($M$w/$M$n) of the polyethylene were determined by a PL-GPC220 (Agilent Technologies Inc., Santa Clara, CA, USA) at 150 °C with 1,2,4-trichlorobenzene as the eluting solvent. Differential scanning calorimetry was employed to measure the melting points of polyethylene. These measurements were obtained from the second scan using a PerkinElmer TA-Q2000 DSC (PerkinElmer Scientific, Waltham, MA, USA), with a heating rate of 10 °C/min. High-temperature $^{13}C$ NMR spectra of the polyethylene samples were recorded on a Bruker AVANCE III 500 MHz instrument (Bruker Corporation, Billerica, MA, USA) at 110 °C in 1,1,2,2-tetrachloroethane-d2 ($C_2D_2Cl_4$) with TMS as an internal standard. In this article, the software of Office 2021, Origin2021, MestReNova 14, Olex2-1.5, and Chemdraw 2020 was used.

## 2. General Procedure for Ethylene Polymerization

The ethylene polymerization was performed in a 250 mL stainless steel autoclave, fitted with a mechanical stirrer and a temperature control system. The autoclave, previously dried in an oven at 100 °C, was evacuated under vacuum. The autoclave was then purged twice with nitrogen, followed by one purge with ethylene, and this process was repeated three times. Subsequently, 25 mL of a toluene containing dissolved nickel precatalyst was introduced into the autoclave containing ethylene (at approximately 1 atm), followed by an additional 25 mL of toluene. The reaction temperature was then elevated by 10 °C above the target polymerization temperature, and the requisite cocatalyst (MAO, MMAO, EASC, or $Et_2AlCl$), along with the remaining 50 mL of toluene, was incrementally added via syringe, bringing the total solvent volume to 100 mL. Ethylene was then supplied at the designated pressure (1/5/10 atm) to initiate polymerization under stirring. Upon completion of the desired reaction period, the system was cooled in a water bath, and ethylene was gradually vented to atmospheric pressure. The remaining solution was quenched with 10% HCl-acidified ethanol, and the precipitated polyethylene was separated by filtration, washed with ethanol, and dried under vacuum at 60 °C until a constant weight was achieved. The resulting polyethylene was then weighed and subjected to characterization.

## 3. X-ray Crystallographic Studies

Single-crystal X-ray diffraction analysis of **Ni4** and **Ni5** was conducted on a Rigaku Saturn 724+ CCD with graphite monochromatic Mo-Kα radiation (λ = 0.71073 Å) at 173.15 K, and the cell parameters were obtained by global refinement of the positions of all collected reflections. The unit cell parameters were determined through global refinement of all collected reflection positions. Intensity data were corrected for Lorentz and polarization effects, as well as empirical absorption corrections. The crystal structures were solved using direct methods and subsequently refined via full-matrix least squares on $F^2$. Hydrogen atoms were positioned at calculated sites. Structure solution and refinement were

performed with the SHELXTL-97 software package[61,62]. Crystal data and crystallographic parameters for **Ni4** and **Ni5** are summarized in Table S1.

**Table S1.** Crystal data and structure refinement for **Ni4** and **Ni5**.

| | **Ni4** | **Ni5** |
|---|---|---|
| Empirical formula | $C_{46}H_{44}Br_2N_2Ni$ | $C_{48}H_{48}Br_2N_2Ni$ |
| Formula weight | 843.36 | 871.41 |
| Temperature/K | 173.15 | 173.15 |
| Crystal system | monoclinic | triclinic |
| Space group | $P2_1/c$ | P-1 |
| a/Å | 9.6197(19) | 9.3401(19) |
| b/Å | 16.993(3) | 12.864(3) |
| c/Å | 24.779(5) | 19.561(4) |
| α/° | 90 | 90.81(3) |
| β/° | 100.28(3) | 94.43(3) |
| γ/° | 90 | 103.11(3) |
| Volume/Å$^3$ | 3985.6(14) | 2281.0(9) |
| Z | 4 | 2 |
| $\rho_{calc}$(g/cm$^3$) | 1.406 | 1.269 |
| μ/mm$^{-1}$ | 2.526 | 2.209 |
| F(000) | 1728.0 | 896.0 |
| Crystal size/mm$^3$ | 0.436 × 0.335 × 0.141 | 0.407 × 0.388 × 0.145 |
| Radiation | MoKα (λ = 0.71073) | MoKα (λ = 0.71073) |
| 2θ range for data collection/° | 4.794 to 54.978 | 2.09 to 54.988 |
| Index ranges | $-12 \leq h \leq 12$, $-21 \leq k \leq 21$, $-32 \leq l \leq 32$ | $-11 \leq h \leq 12$, $-16 \leq k \leq 16$, $-25 \leq l \leq 25$ |
| Reflections collected | 26876 | 29809 |

| | | |
|---|---|---|
| Independent reflections | 9068 [$R_{int}$ = 0.0457, $R_{sigma}$ = 0.0496] | 10401 [$R_{int}$ = 0.0597, $R_{sigma}$ = 0.0547] |
| Data/restraints/parameters | 9068/0/462 | 10401/1/480 |
| Goodness-of-fit on $F^2$ | 1.114 | 1.046 |
| Final R indexes [I>=2σ (I)] | $R_1$ = 0.0564, $wR_2$ = 0.1197 | $R_1$ = 0.0509, $wR_2$ = 0.1372 |
| Final R indexes [all data] | $R_1$ = 0.0663, $wR_2$ = 0.1251 | $R_1$ = 0.0588, $wR_2$ = 0.1430 |
| Largest diff. peak/hole / e Å$^{-3}$ | 0.70 and –0.73 | 0.51 and –0.60 |

## 4. $^1$H NMR spectra of imino-ketone and ligands (L1-L5)

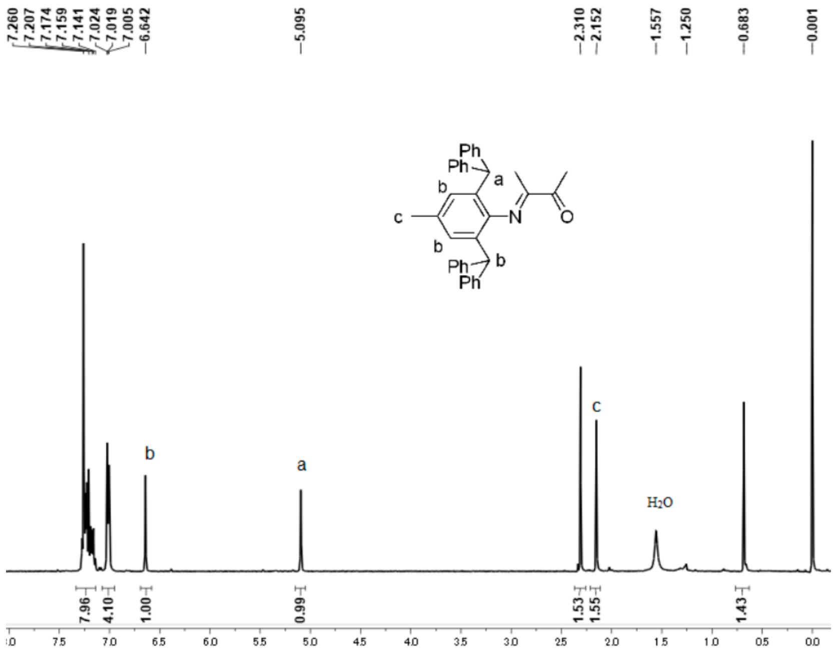


**Figure S1.** $^1$H NMR (400 MPa) spectrum of 2-(2,6-dibenzhydryl-4-methylphenylimino)butanone (recorded in $CDCl_3$ at 25 °C).

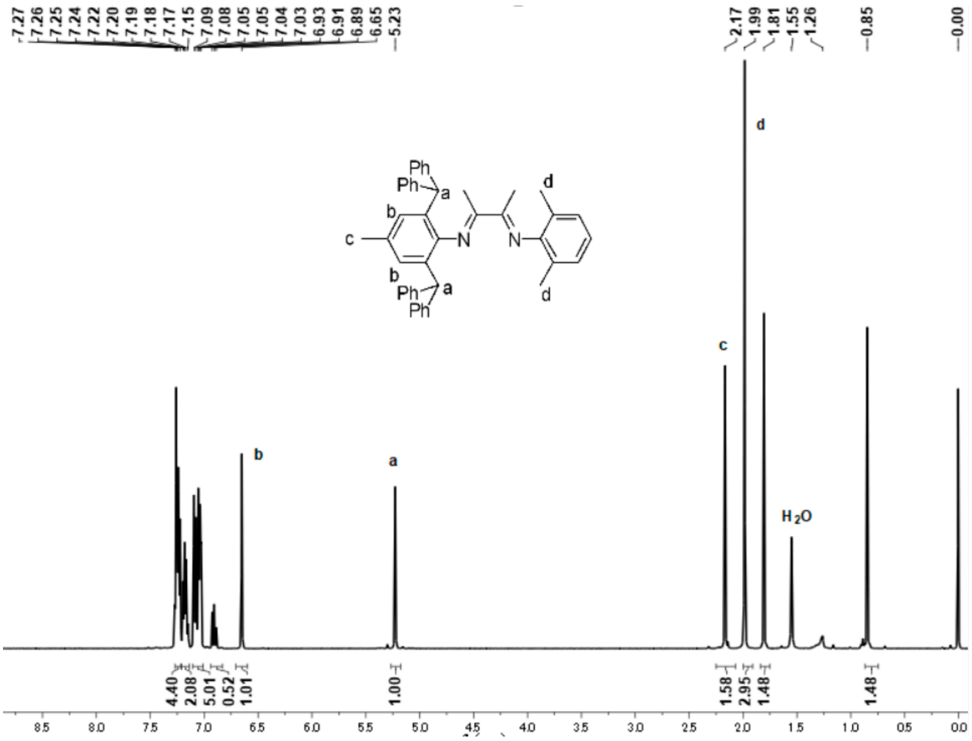


**Figure S2.** $^{1}$H NMR (400 MPa) spectrum of **L1** (recorded in $CDCl_3$ at 25 °C).

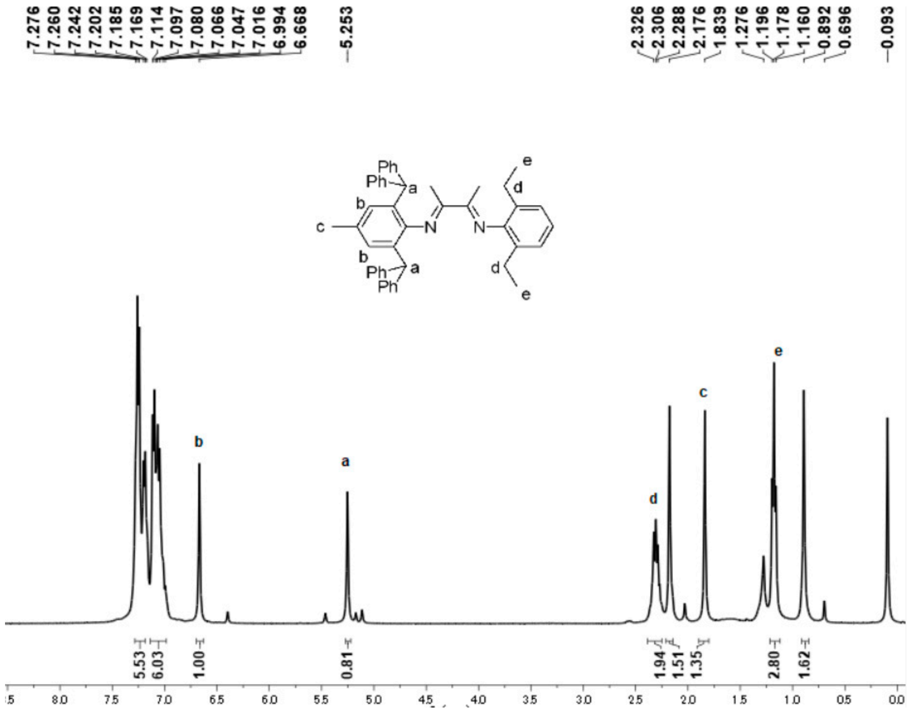


**Figure S3.** $^{1}$H NMR (400 MPa) spectrum of **L2** (recorded in $CDCl_3$ at 25 °C).

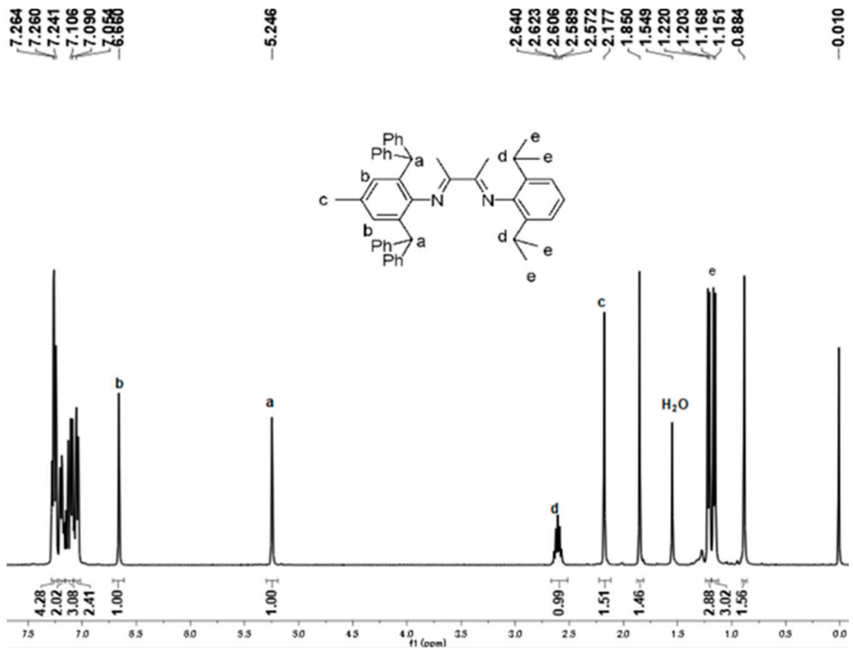


**Figure S4.** $^{1}$H NMR (400 MPa) spectrum of **L3** (recorded in $CDCl_3$ at 25 °C).

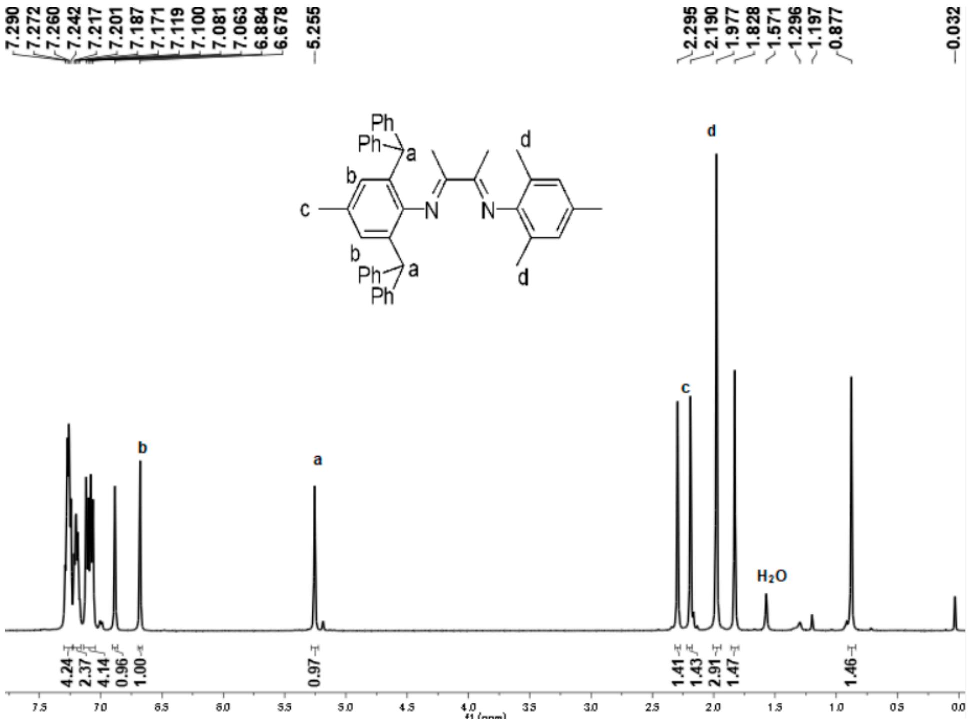


**Figure S5.** $^{1}$H NMR (400 MPa) spectrum of **L4** (recorded in $CDCl_3$ at 25 °C).

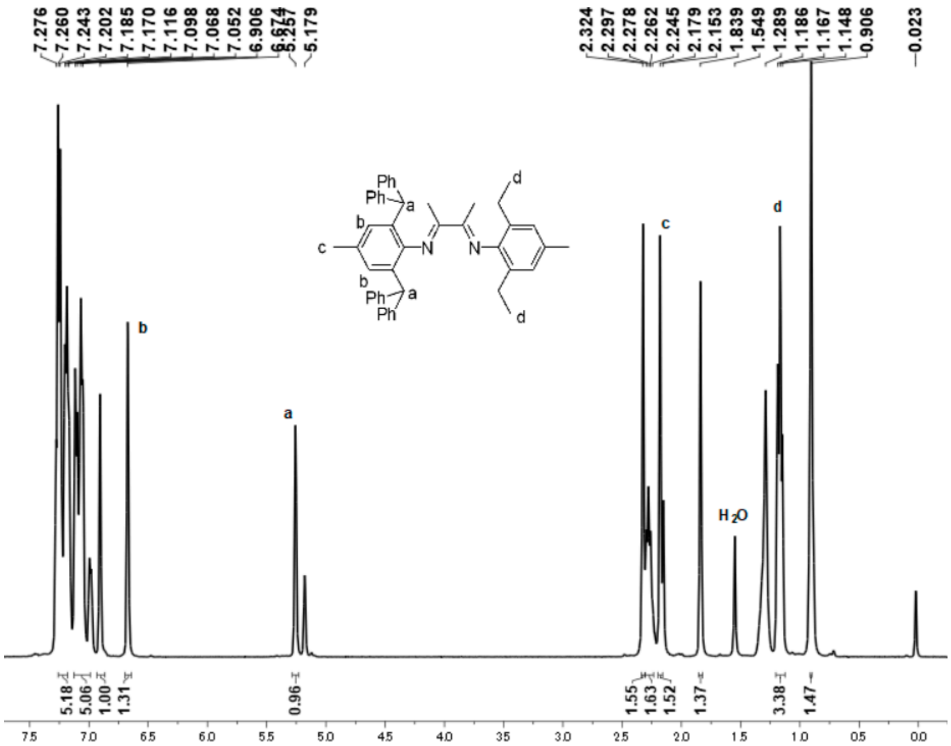


**Figure S6.** $^{1}$H NMR (400 MPa) spectrum of **L5** (recorded in $CDCl_3$ at 25 °C).

## 5. References

[61] Sheldrick, G. SHELXT-Integrated space-group and crystal-structure determination. *Acta Crystallographica Section A* **2015,** 71, 3–8.

[62] Sheldrick, G. Crystal structure refinement with SHELXL. *Acta Crystallographica Section C* **2015,** 71, 3–8.
